# Supplementary material for: Patient preferences when searching for clinical trials and adherence of study records to ClinicalTrials.gov guidance in key registry data fields
Source: PLoS One. 2020 May 29;15(5):e0233294. doi: 10.1371/journal.pone.0233294 (PMC7259626; doi:10.1371/journal.pone.0233294)
Supplement: S1 Appendix — (PDF) [file pone.0233294.s001.pdf]

TransCelerate BIOPHARMA INC.

ACCELERATING THE DEVELOPMENT OF NEW MEDICINES

Clinical Register Data Packet (CRDP)

2019 Global Survey Findings

Have you ever participated in a clinical trial?

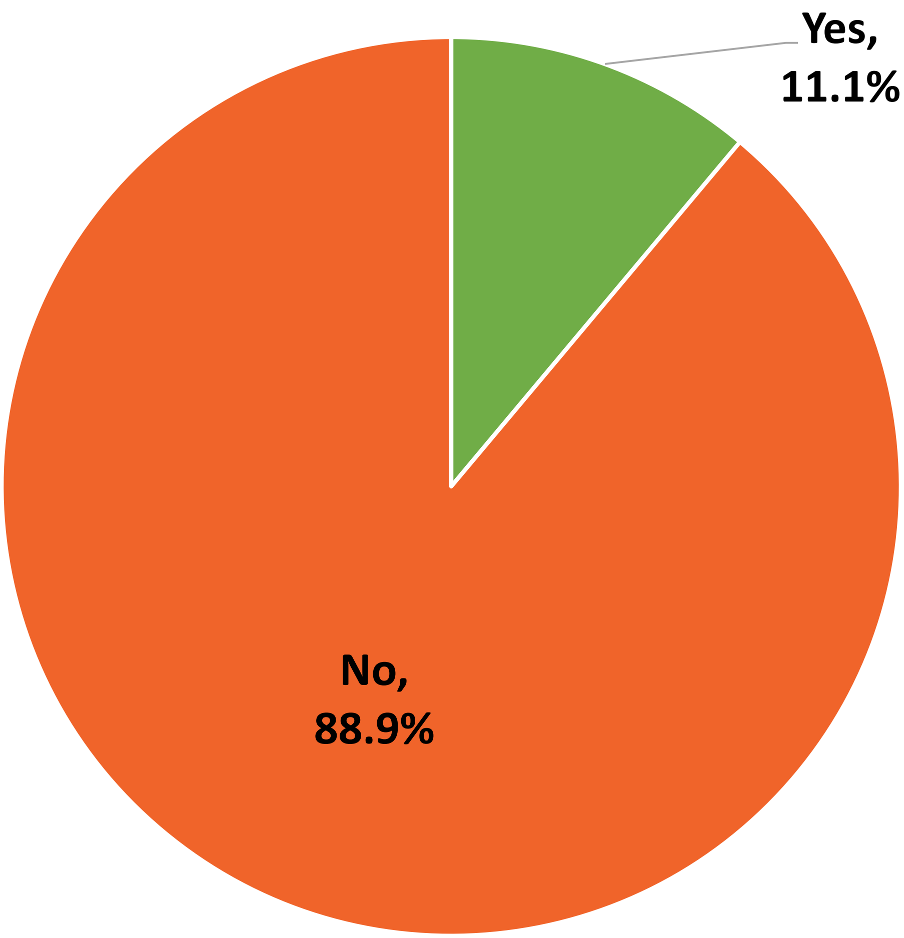

Total Responses: 1,070

How did you first learn of your clinical trial?

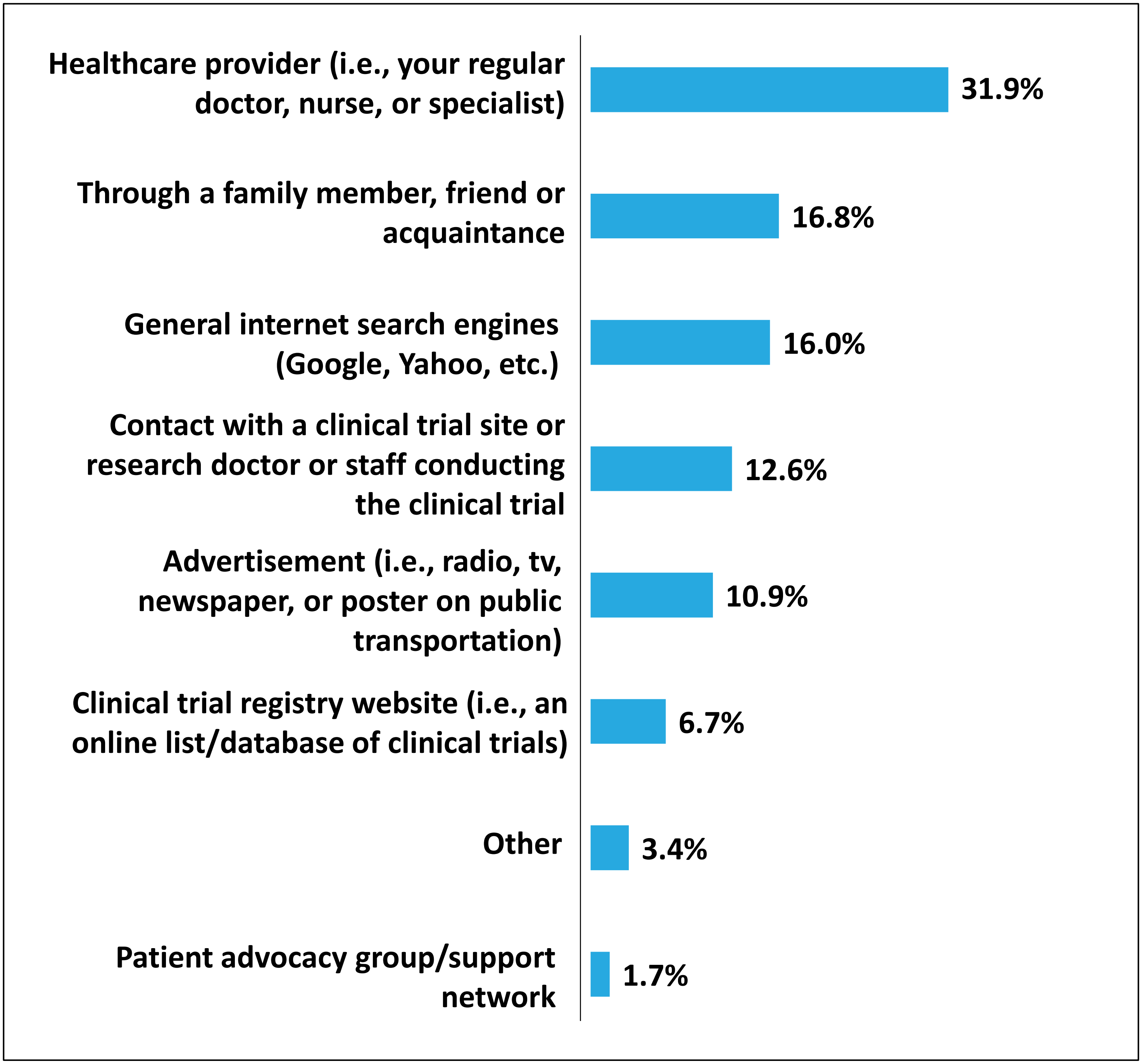

Total Responses: 119, those that participated in clinical trial

Have you ever visited a clinical trial registry website?

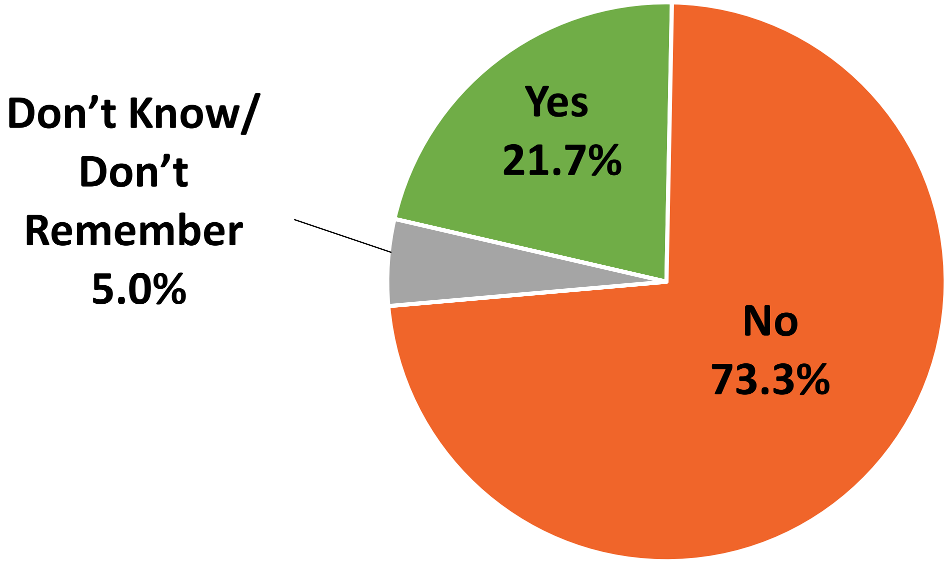

Total Responses: 1,070

If yes, which one did you visit?

Name of Site	% of Respondents Selecting	Number of Respondents Selecting
National Health Institute (NIH) ClinicalTrials.gov	51.3%	119
Pharmaceutical company clinical trial registry	38.8%	90
World Health Organization (WHO) International Clinical Trials Registry Platform (www.who.int)	28.9%	67
I I do not remember which one	24.6%	57
European Union Clinical Trials Register (www.clinicaltrialsregister.eu)	23.7%	55
Other	3.0%	7

Total Responses: 232

Are you aware that sponsors (i.e. organizations that fund the clinical trial) sometimes ask patients for input to help design the clinical trial?

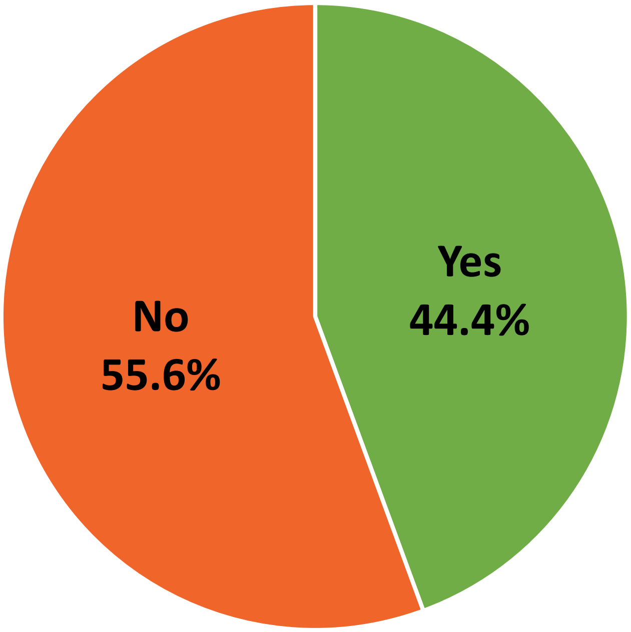

Total Responses: 1,070

If yes, have you ever been asked for input to help design a clinical trial?

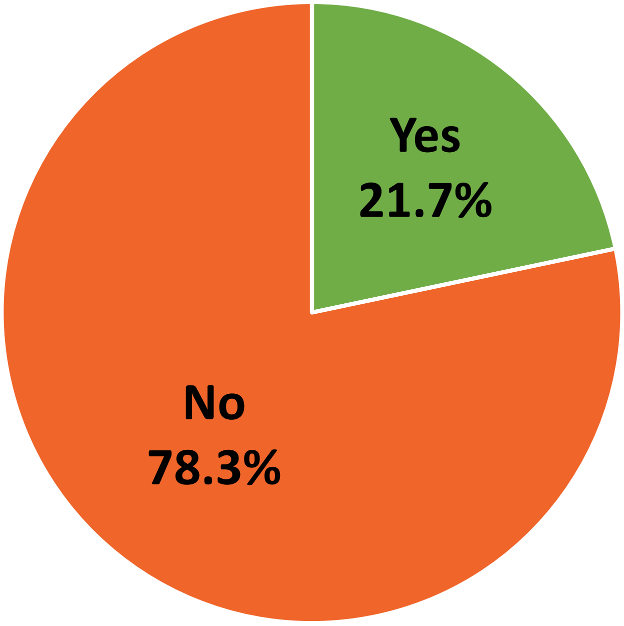

Total Responses: 475

Imagine you're visiting a clinical trial registry website to search for clinical trials.

What kind of information would you find most helpful when you begin looking for clinical trials?

Information Type	% of Respondents Selecting	Number of Respondents Selecting
Condition or disease being studied in the clinical trial	66.4%	711
Location of the trial	57.0%	610
The dates the clinical trial will start and end	52.9%	566
Age and gender of participants	48.6%	520
Health measurement(s) or observation(s) examined to determine the effect from the clinical trial drug	45.5%	487
Name of the clinical trial drug being studied	42.5%	455
Study type (Interventional vs. Observational)	38.4%	411
Whether or not the clinical trial is actively enrolling new study volunteers	38.2%	409
Phase of the clinical trial	32.4%	347
Whether or not participants will be able to continue using the clinical trial drug after the clinical trial ends	28.5%	305
Information on who is funding the clinical trial	25.9%	277
Key words describing the clinical trial	23.6%	252

Total Responses: 1,070

Now imagine that you've completed a search and have a list of clinical trials. Some clinical trial information are fields entered by sponsors as free text and others are drop down or pick list fields. The free text fields are below. Rank the free text fields from most (top) to least (bottom) helpful when evaluating clinical trials.

Information Type	Overall Rank	Score
Condition or disease being studied in the trial	1	6,587
A brief summary that provides a general overview of the clinical trial, written in plain language	2	6,242
Detailed information about the clinical trial drug being studied	3	5,825
A short title describing the clinical trial, written in plain language	4	5,675
Criteria needed for participation, for example inclusion/exclusion criteria	5	5,531
Health measurement(s) or observation(s) examined to determine the effect from the clinical trial drug	6	5,428
Location of where the trial will be conducted	7	5,266
Chance of receiving the clinical trial drug if there is a placebo	8	3,925
Information on who is funding the clinical trial	9	3,671

Total Responses: 1,070

What kind of information would be helpful for you to first see in a short title of a clinical trial?

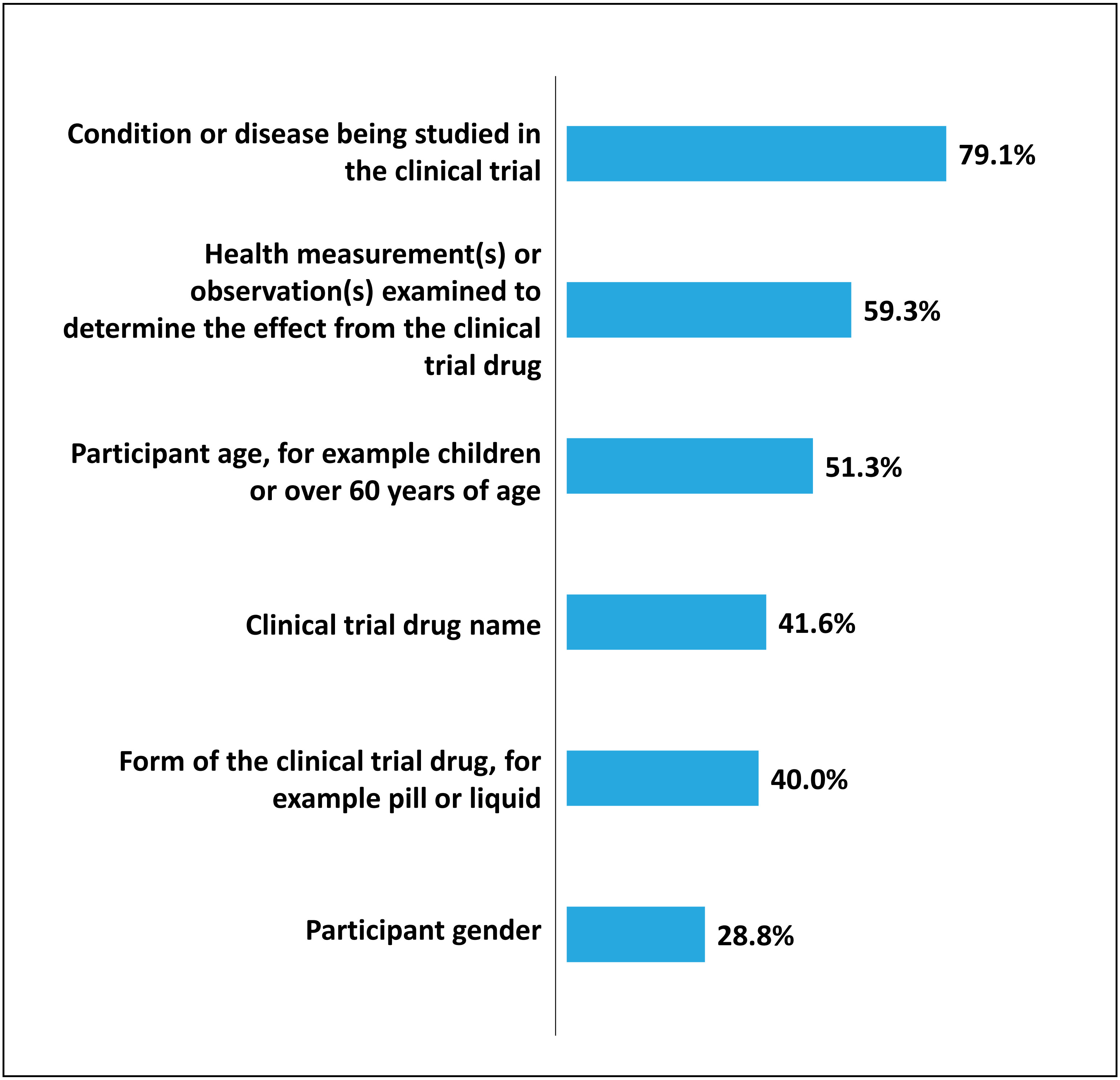

Total Responses: 1,070

What kind of additional information would be helpful to see in a brief summary of a clinical trial?

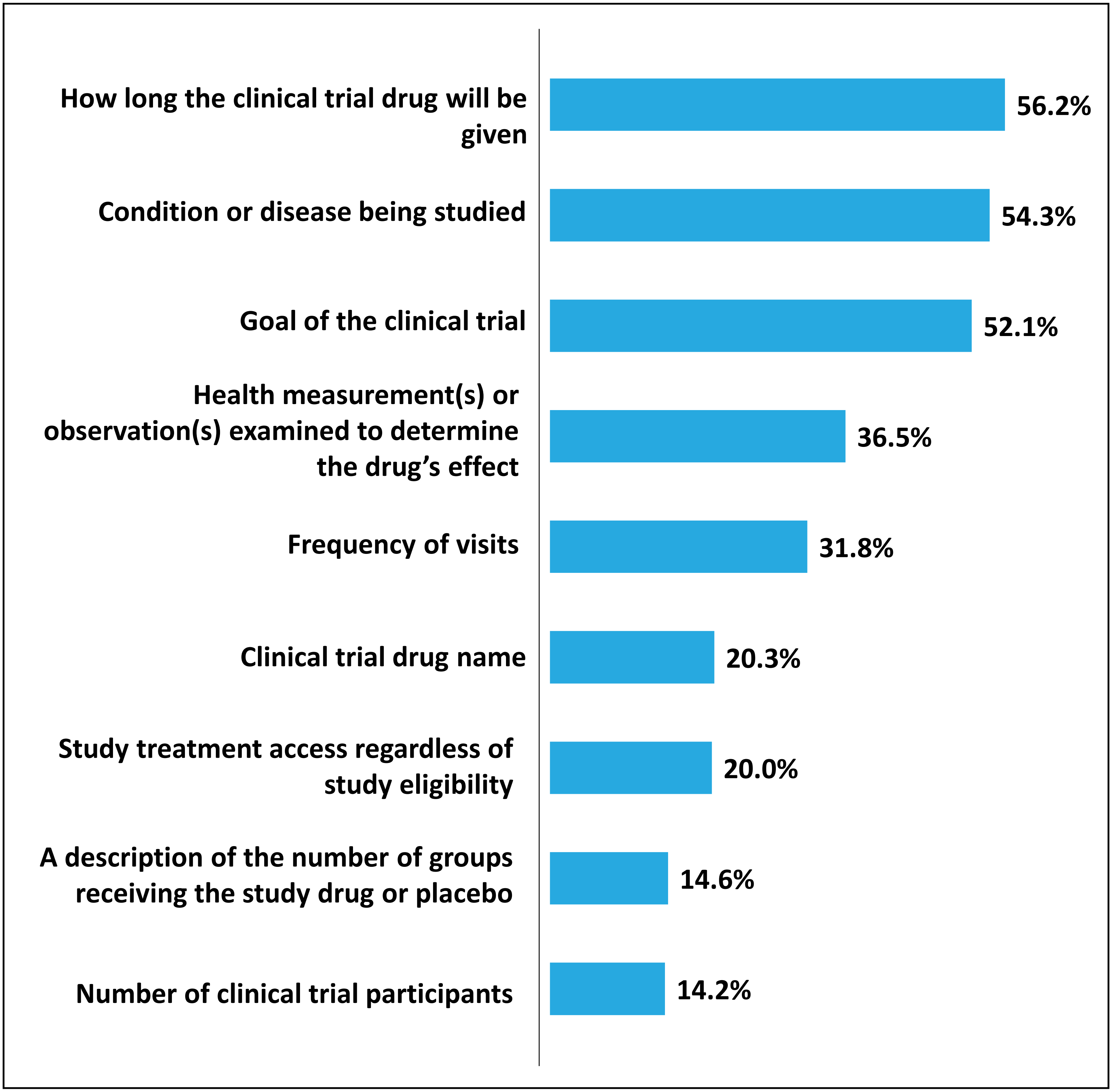

Total Responses: 1,070

The following information is typically made available about a clinical trial drug in listings for clinical trials.

Rate the following pieces of information each on a scale from 'Not at all helpful' to 'Very helpful' when evaluating a clinical trial.

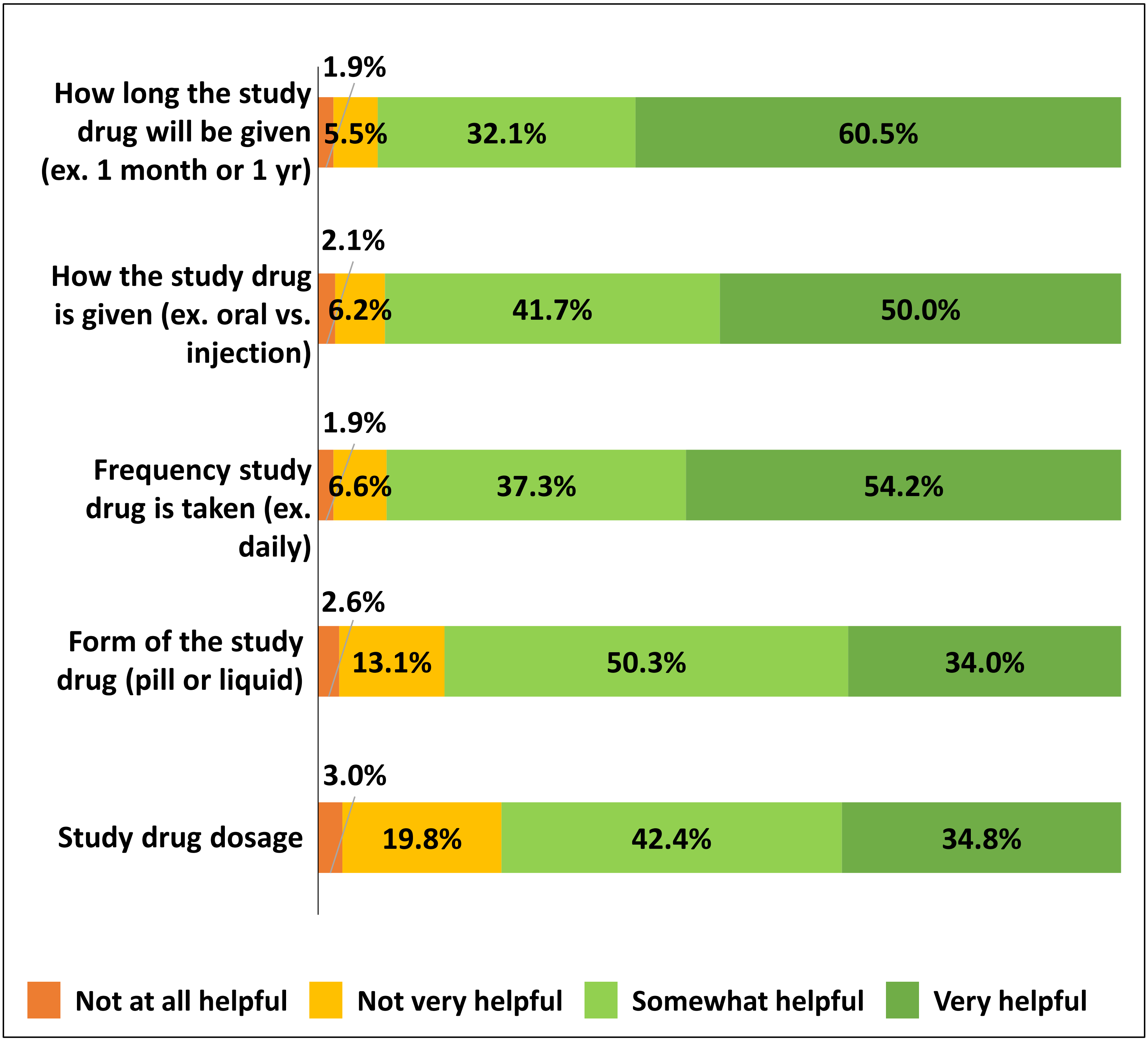

Total Responses: 1,070

When looking at the inclusion/exclusion criteria, which would you prefer to see?

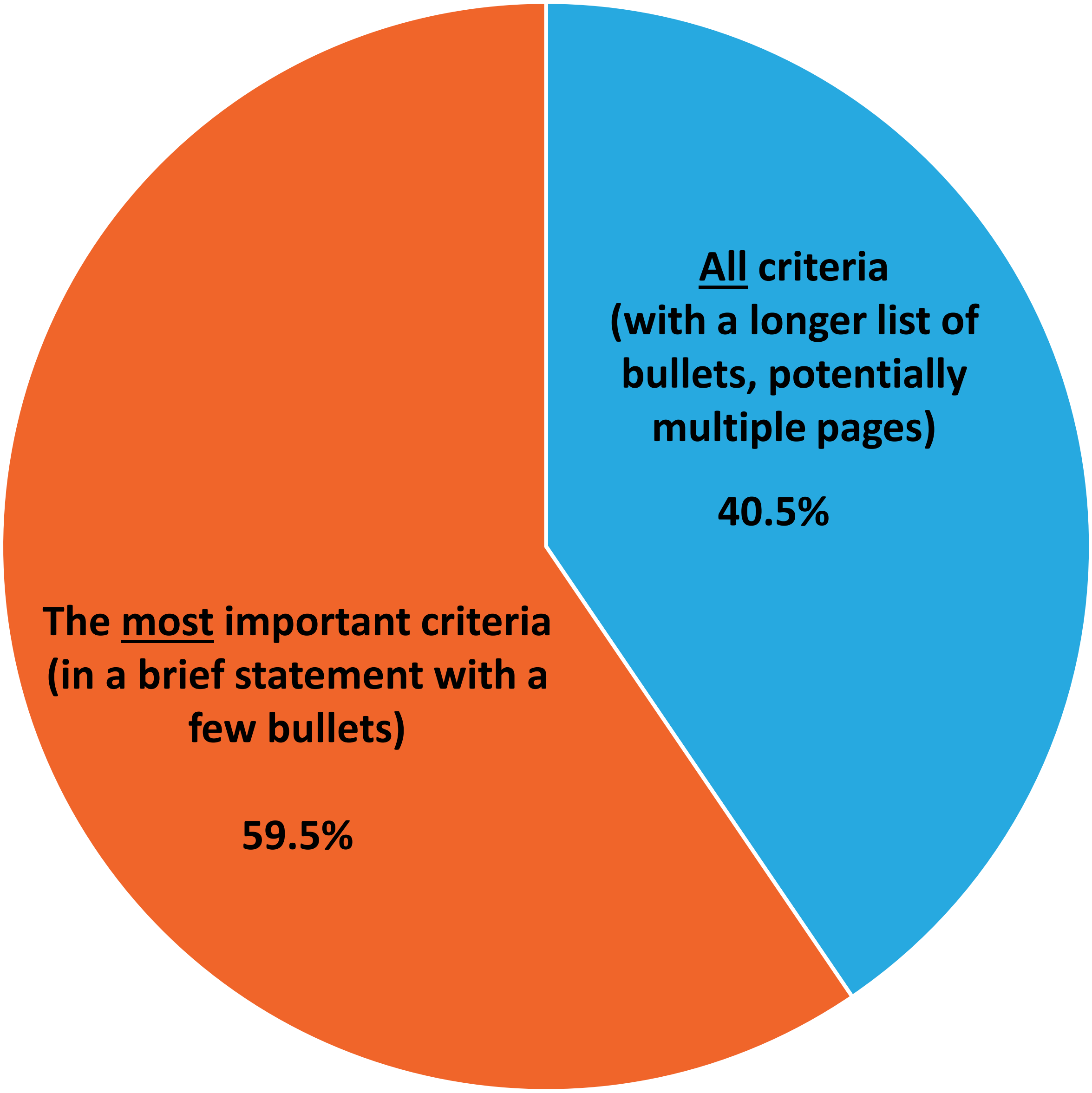

Total Responses: 1,070

Demographics

What country do you reside in?

Country	Percent	Number of Responses
**United States**	25.6%	274
**Japan**	20.8%	223
**Australia**	12.5%	134
**United Kingdom**	10.9%	117
**Germany**	9.6%	103
**Spain**	7.5%	80
**Mexico**	7.0%	75
**All others**	6.2%	64

Total Responses: 1,070

Demographics (cont.)

Which of the following best describes your gender?

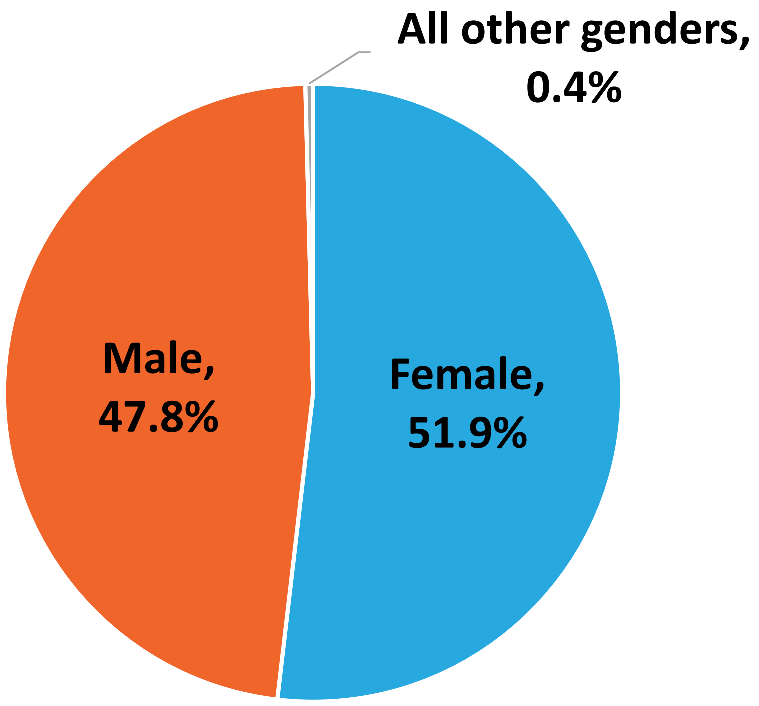

How old are you?

Min. Age	Max. Age	Average Age	StdDev
18 years old	76 years old	45 years old	14.2

Total Responses: 1,070

Demographics (cont.)

What is the highest level of education you have completed?

Primary or less	High school (some or diploma)	Technical training	Some college	Bachelor's Degree	Master's Degree	Doctorate/ professional
1.3%	24.1%	13%	19%	23%	11.7%	8.1%

How would you describe your employment status?

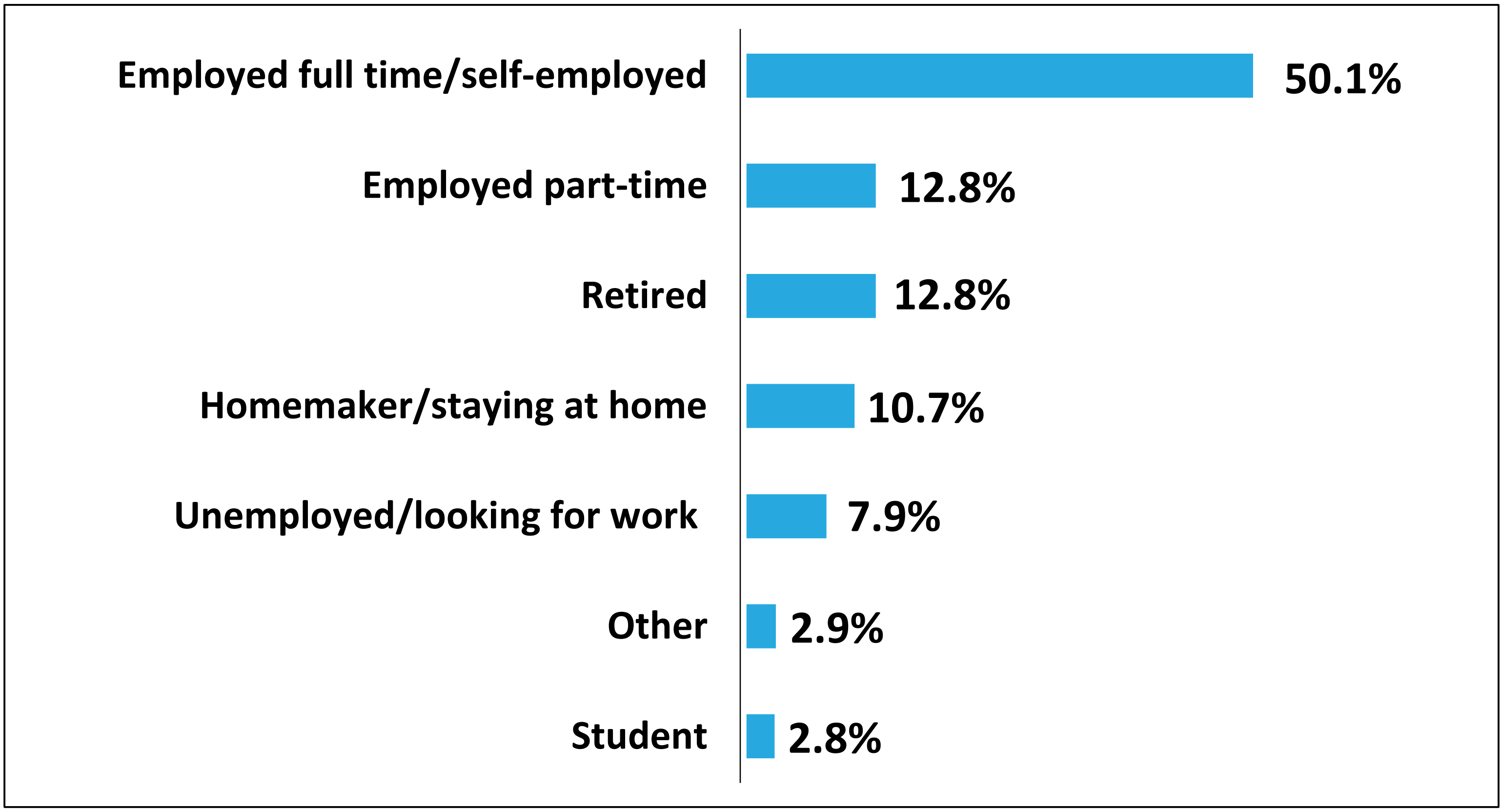

Total Responses: 1,070
