# Supplementary material for: Patient preferences when searching for clinical trials and adherence of study records to ClinicalTrials.gov guidance in key registry data fields
Source: PLoS One. 2020 May 29;15(5):e0233294. doi: 10.1371/journal.pone.0233294 (PMC7259626; doi:10.1371/journal.pone.0233294)
Supplement: S2 Appendix — (PDF) [file pone.0233294.s002.pdf]

ClincialTrials.gov Data Field Analysis Guide

Brief Title

Assess the Brief Titles delegated to you from ClinicalTrials.gov for the presence/absence of the following information across the categories of format and content. Where needed, reference information from other data fields such as 'Condition' or 'Intervention' on the spreadsheet if you're unclear on what may or may not be present in a given Brief Title. Keep in mind that this is not a consistency check exercise across data fields.

Format (2 areas)

1) **Periods**
   - Enter "**1**" for a "**YES**" if a period is <u>absent</u> at the end
   - Enter "**0**" for a "**NO**" if a period is <u>present</u> at the end
2) **Abbreviations**
   - Enter "**1**" for a "**YES**" if:
     - All abbreviations included are defined
     - No abbreviations are used
   - Enter "**0**" for a "**NO**" if:
     - An abbreviation is included but is not defined
     - Abbreviations are included but all are not defined

Content (5 areas)

3) **Condition:** Does the brief title indicate the heath condition(s) being assessed by the trial (e.g. diabetes, heart disease)?
   - Enter "1" for a "YES" if:
     - The brief title makes any mention of the condition being studied.
     - If the condition is abbreviated, regardless of whether the abbreviation is not defined or not.
   - Enter "0" for a "NO" if the condition is absent
4) **Information on the participants:** Does the brief title indicate that participants are required for the trial and, in a sense, humanize the brief title? (6 parts)
   1. Use of the term 'Patient'
      - Enter "**1**" for a "**YES**" if 'Patient' is present
      - Enter "**0**" for a "**NO**" if 'Patient' is absent
   2. Use of the term 'Participant'
      - Enter "**1**" for a "**YES**" if 'Participant' is present
      - Enter "**0**" for a "**NO**" if 'Participant' is absent
   3. Use of the term 'Participant'
      - Enter "**1**" for a "**YES**" if 'Volunteer' is present
      - Enter "**0**" for a "**NO**" if 'Volunteer' is absent
   4. Use of the term 'Volunteer
      - Enter "**1**" for a "**YES**" if 'Subject' is present

        - Enter “**0**” for a “**NO**” if ‘Subject’ is absent
    5. Participant age
        - Enter “**1**” for a “**YES**” if age is present in either numerical range (e.g. over 50 years of age) or categorical (e.g. adult, pediatric) forms
        - Enter “**0**” for a “**NO**” if age is absent
    6. Participant gender/sex
        - Enter “**1**” for a “**YES**” if gender/sex is present e.g. men, women, males, etc
        - Enter “**0**” for a “**NO**” if gender/sex is absent

5) **Intervention(s)**: Does the brief title provide information on the intervention(s) being studied? If more than one drug is mentioned, then the assessment is limited to the study drug(s), not the comparator. (5 parts)
    1. Intervention name
        - Enter “**1**” for a “**YES**” if ANY proprietary, scientific, generic or brand name of the study drug is present, even if abbreviated (e.g. BMC-986567, insulin)
        - Enter “**0**” for a “**NO**” if any intervention name is absent
    2. Form of the interventional drug
        - Enter “**1**” for a “**YES**” if the form of the study drug is present
            - Examples of administration include: *tablet, pill, capsule, patch, cream, ointment, injection*
        - Enter “**0**” for a “**NO**” if the form of the study drug is absent
    3. Route of administration
        - Enter “**1**” for a “**YES**” if how the study drug is administered is present
            - Examples of administration include: *oral, topical, intravenous*
        - Enter “**0**” for a “**NO**” if the route of study drug administration is absent

6) **Health measurements:** The health measurement[s] or observation[s] examined to determine the effect from the trial drug (e.g. the reduction in bad cholesterol, kg of weight loss). The language can be similar to the study objectives or primary outcome measures (see the additional columns of data field information on the spreadsheet).
        - Enter “**1**” for a “**YES**” if any health measurements are present
            - Terms that are common to health measurements: *reduction, correction, reversal, lower, increase, improve*
        - Enter “**0**” for a “**NO**” if any health measurements are absent
            - Terms that may seem like health measurements but are <u>not</u>: *safety, efficacy, evaluate*

7) **Technical study design terms:** Technical study design terms describe the design/execution of a study. NIH *discourages* the use of technical study design terms in Brief Titles, so we are scoring titles without such terms as “1”. Keep in mind that technical study design terms do not include other technical terms that may describe interventions, eligibility criteria, primary outcome measurements, etc.
        - Enter “**1”** for a “**YES**” if technical design study terms are <u>absent</u>
            - Terms that may seem like technical study design terms but are <u>not</u>: *placebo, first-line treatment, topical, intravenous, symbols (e.g. <, >, ≥), safety, efficacy, immunogenicity, the name of an intervention (e.g. BS478)*

- Enter “**0**” for a “**NO**” if one or more technical study design terms are present based on your opinion. In the column titled ‘Technical study design terms present’, write down the technical study design terms you identified in each Brief Title.
    - Examples of technical study design terms (not exhaustive): *Phase 2, single group, double blind, randomized, pharmacokinetics, non-inferiority trial, multicenter*
    - For more examples see section ‘7. Study Design’ on https://prsinfo.clinicaltrials.gov/definitions.html
