# Supplementary material for: Patient preferences when searching for clinical trials and adherence of study records to ClinicalTrials.gov guidance in key registry data fields
Source: PLoS One. 2020 May 29;15(5):e0233294. doi: 10.1371/journal.pone.0233294 (PMC7259626; doi:10.1371/journal.pone.0233294)
Supplement: Attachment — Submitted filename: Response to reviewers.docx [file pone.0233294.s003.docx]

5. Review Comments to the Author

Please use the space provided to explain your answers to the questions above. You may also include additional comments for the author, including concerns about dual publication, research ethics, or publication ethics. (Please upload your review as an attachment if it exceeds 20,000 characters)

**Reviewer #1**: As the authors point out, the information contained in glinicaltrials.gov should be adapted to the needs of the patients. There are no previous studies that have evaluated this aspect, so the work is relevant, very current and may have implications for improving the information in the database.

The project, divided into several phases, has generated some interesting results. However, in my opinion, neither the structure of the work nor its contents follow the basic rules of an original article. The work has more the structure of a "report" than of a scientific article. Although it is structured in the classic sections of “introduction, material and methods”, “discussion” and “conclusions”, each of these asections mixes aspects of the rest.

**Author reply**: The manuscript is reporting research on an aspect of clinical trial registries that has received no attention to date. The research has taken more than 3 years and was designed as a sequential investigation meaning that the results of the initial phase such as the patient advisory boards have supported the design of the survey whose results has then helped the analysis of the current status of ClinicalTrials.gov entries. This sequential approach might be perceived as a “report” but we do not share the concern that providing the intermediate results is not scientific. This notwithstanding, we have followed the reviewer’s suggestion wherever possible.

**Reviewer #1**: Objectives: the main objectives of the study must be clearly specified at the end of the introduction, avoiding the inclusion of aspects of the methodology (first paragraph on page 5).

**Author reply**: The text was changed and the main objectives were stated at the end of the introduction.

**Reviewer #1**: Methods: At the beginning of this section, the authors should give a general perspective on the methodology used, indicating the primary, secondary objectives, as well as all the analyzes that will be carried out, the rating scales included, the criteria used etc. The Validation Assessment section includes “results” that should go to the corresponding section.

**Author reply**: As per request, a general section was introduced at the beginning of the methods section. We did however not include a description of the rating scales in the methods section because these scales were developed as a result of the PAB and the survey, ie constitute an intermediate result and not a method. Therefore, it is not useful to introduce the scales in the methods section as this would confuse the reader. Contrary to the reviewer’s suggestion, the section Validation Assessment was not changed as the result component in this paragraph is limited to a single sentence. Moving this sentence to the results section would have needed to repeat the presentation of the methods which would have made the paper even longer and more complex.

**Reviewer #1**: Results: the reading of this section is extremely complex, excessively fragmented and it is not obvious which are the most relevant findings. In addition, each of the sections begins with a methodological description that was not previously specified in the "Methods" section. For example, the last paragraph on page 13, the last paragraph on page 14, the first paragraph on page 16, or the last paragraphs on pages 17 and 18 describe for the first time the methods used, the scales applied, etc.

**Author reply**: The reviewer is using page numbers to refer to several sections. We have tried to identify these sections based on the page numbers in the pdf version in the PLOS ONE publishing tool. We are however not always sure to have determined the right sections. A mismatch could have occurred if the paper was printed on US standard size and this was based for the reviewers comments.

We largely followed the reviewer’s suggestion to include more detail into the methods section. We moved most of the first paragraph on page 16 starting with “Using” and ending with “criteria” to the methods section. In addition, we also moved last section on page 18 starting with “As a second” and ending with “format” on page 19 together with the first paragraph in the section entitled “Guidance adherence assessment of brief titles” to the method section. We also moved the last paragraph on page 20 starting with “ClinicalTrials.gov provides” and ending on page 21 with (Table 7)” to the methods section.

We could however not follow the other suggestions, in detail: The last paragraph on page 13 starting with “Survey participants” and ending with “(Table 3)” describes a subsequent question in the survey and introduces the responses to this question in the global online survey. This is not a description of a new method. The paragraph needs to stay to maintain the flow in the description of the results.

The same applies to the last paragraph on page 14 starting with “Two questions” ending with (Fig.1)”. The text simply introduces the results to the next question in the survey and does not describe a method. Therefore we did not change the text.

The last paragraph on page 17 starting with “Based on our” and ending with “format” does also not describe a methodology but describes the results of the patient focus assessment for brief summaries and was therefore not changed.

**Reviewer #1**: Tables 4 and 5 are not easy to interpret and their contents do not seem to be aligned with the results of the text.

**Author reply:** The section referring to Table 4 was revised for better flow, we did however not find any discrepancies between the text and the content of the table.

**Reviewer #1**: In my opinion, the work should include fewer tables and figures, selecting the most relevant findings of the work. Although they include different analyses, some of the results in Tables 2-4 seem contradictory. For example, “location” appears as one of the most important items in table 2 and occupies a lower place in table 3. This type of inconsistencies should be explained in the Discussion.

**Author reply**: We have carefully assessed the number of tables in the manuscript. There are a total of 8 tables and 2 figures. Each of the tables represents the results of an individual analysis and there is no duplication in content. We believe that all tables are needed to fully inform the readers about the results.

We checked Tables 2 -4 and did not identify any conflicting information. Table 2 provides the patient preferences when patients are starting to look for a clinical study, ie the items they believe are most important in identifying the appropriate studies. This is simulating a situation of a patient when starting to create the search terms with which she is trying to identify the studies of interest. Table 3 provides the preferences of patients when they are selecting a trial from a list that is returned by the registry, ie an initial search result. Tables 4 is unrelated to the previous tables as it reflects the result of our analysis of current ClinicalTrials.gov entries. In summary, the tables reflect different situations and their content is therefore not conflicting. The

**Reviewer #1**: The reason for the diversity in the rating scales and the criteria used should be explained: some range from 1 to 7, others from 1 to 8, figures 1 and 2 do not use the same credentials (one of the three most helfulp vs the item is helpful). The reasons for these variations should be explained.

**Author reply**: The differences in the rating scale originates in their different purposes. The rating scale for patient focus of brief titles consists of 7 items which were derived from the patient survey and the feedback from the patient advisory boards. Therefore, the scale ranges from 1 to 7. The rating scale for patient focus of brief summaries consists of 8 items which were also derived from the online survey and the feedback from the patient advisory boards, therefore the scale ranges from 1 to 8. There is no variation as the scales are applied to different entities namely the brief title and the brief summary.

**Reviewer #1**: Discussion: The discussion must interpret the results, however, the authors repeat again the objectives (last paragraph of page 23, second paragraph of page 24) and the methods (first paragraph of the discussion, third paragraph of page 24, second paragraph on page 25 etc.). The most important results should be discussed and interpreted, emphasizing what the study provides, the most relevant results, the most surprising, the limitations, etc.

The main results (first paragraph on page 25) should be commented at the beginning of the discussion and not on the third page. The first 3-4 paragraphs of the discussion are repetitive and do not constitute a discussion on the subject.

It seems that one of the conclusions of the study is that a short title should include information on: the condition, health measures (??), the age of the participants, the name of the drug, and the form of the drug. Is this really the relevant information for patients? Do authors think that information on the health measures used contributes more to patients engagement in research than information on start and end dates of the study? These are somewhat surprising results that the authors should comment on the discusion.

**Author reply**: The discussion was rewritten to accommodate the comments of the reviewer. The question on whether the authors find “the condition, health measures (??), the age of the participants, the name of the drug, and the form of the drug” relevant is surprising because the purpose of our research was to find out what patients want. To determine this, we conducted the survey and the patient advisory board meetings. The question whether the authors believe the information is relevant misses the whole point of our research.

**Reviewer #1**: Conclusion: It is not clear which are the 2-3 main conclusions of the study.

**Author reply**: We believe the conclusions are clearly stated, namely that brief title and brief summary are important data fields for patients and other registry users to identify suitable studies. We also conclude that the current entries in ClinicalTrials.gov reflect a tendency of sponsors to put more emphasis on adherence to guidelines than on patient focus thus missing the initial intention of ClinicalTrials.gov.
